# Sulphites in Tonsilitis

**Published:** 1873-04

**Authors:** 


Sulphites in Tonsilitis.—Dr. Thomas A. Elder thus speaks of their use in the *Chicago Medical Journal:*

"I have not met with a single case which the sulphites, administered in sufficiently large and often repeated doses, would not promptly relieve and cure. The doses which I use are those recommended by Dr. Tyrrell, gr. xx. to xxx., repeated every hour, for an adult, and correspondingly large doses for children. The fever is generally dissipated in twelve hours—rarely continues twenty-four. The soreness of throat, headache, etc., are generally as promptly relieved, and forty-eight hours are sufficient for a cure. In children, when saturated, they have produced sweating, and the peculiar cadaveric hue of sulphurous acid fumes.

"When the disease has progressed to the stage of exudation—when the shoe-peg points begin to appear, or later—I have never met with a case which I thought was benefited by the sulphites. I am then accustomed to rely upon a prescription of Prof. Miller, for diphtheria, which has almost invariably given prompt and permanent relief:

"'℞. Morphia muriat., gr. ij.; acid. muriat., dil., tr. ferri chloridi, aa. ʒij.; syrupi, ℥ss.; aquæ destillat., ℥ij. M. Sig. Dose, a teaspoonful three or four times a day, after water.'"

He also highly recommends bisulphite of soda in scarlatina.—*Michigan University Medical Journal.*

